# The Weighted Toxicity Score: Confirmation of a Simple Metric to Communicate Toxicity in Randomized Trials of Systemic Cancer Therapy

**DOI:** 10.1093/oncolo/oyad192

**Published:** 2023-07-14

**Authors:** Jacob Jordan, Robert G Maki

**Affiliations:** Perelman School of Medicine, University of Pennsylvania, Philadelphia, PA, USA; Perelman School of Medicine, University of Pennsylvania, Philadelphia, PA, USA; Abramson Cancer Center, University of Pennsylvania, Philadelphia, PA, USA; Department of Medicine, Memorial Sloan Kettering Cancer Center, New York, NY, USA

**Keywords:** weighted toxicity score, PRO-CTCAE, clinical trial endpoints, adverse events

## Abstract

**Introduction:**

FDA’s Project Optimus was developed in part to better identify appropriate dose and schedule of cancer therapeutics. The tabular method to summarize patients’ maximum toxicity in a clinical trial does not allow for ready comparison to the treatment’s benefit. In this manuscript, we apply a simple tool, the weighted toxicity score (WTS), to trials involving lung cancer immunotherapy and chemotherapy, as well as those cited in a recent publication as examples of trials that represent successful reduction of the appropriate dose of anti-cancer agents.

**Methods:**

PubMed was queried for randomized controlled trials of therapy involving immune checkpoint inhibitors in lung cancer. Trial data from studies highlighting initial success with dose adjustments after FDA approval also were assembled and analyzed according to the WTS procedure described previously, compared to clinical outcomes data.

**Results:**

The WTS provided, with the clinical outcome(s), a data pair that leads to easy interpretation of the expected benefit versus relative toxicity of studies involving immunotherapy or chemoimmunotherapy in lung cancers. The WTS was consistent with the conclusions of the primary studies, helping to quantitate the toxicity difference between treatments in a previously unavailable way.

**Conclusion:**

The WTS provides a tool to show the cost in toxicity of therapy in a randomized clinical trial, with applicability to studies involving chemotherapy, immunotherapy, or kinase-directed therapy. Inclusion of a running tally of WTS during conduct of a trial could serve as one means to adjust dosing or to provide feedback during data safety monitoring of a clinical trial.

## Teaser

The weighted toxicity score provides a rapid way to compare toxicity between 2 arms in a clinical trial. It may help clinicians better define the cost-benefit ratio of specific treatments, a goal of FDA’s Project Optimus. It could also be used prospectively to help assess toxicity on an ongoing basis while a cancer clinical trial is being conducted.

**Implications for Practice**

The weighted toxicity score (WTS) summarizes the toxicity of a treatment used in a randomized cancer clinical trial as a single number and requires no more than the ability to add and multiply. This value allows for a graphical comparison of costs and benefits of treatment and makes it easier to discuss the risks versus outcomes of a given therapy for a particular clinical situation.

## Introduction

The dose and schedule for many anti-cancer agents are defined by the dose-limiting toxicity and maximum tolerated dose derived from phase I clinical trials, with confirmation in later stage testing. Alkylating agents, such as cisplatin, appear to have a dose-response relationship based on its peak level, which had a prolonged impact on how cancer drugs are developed. However, it is increasingly recognized that for many anti-cancer agents, maximum dose intensity is not necessarily associated with greater cancer control, although toxicity is often greater at higher dose intensity. PD1/PD-L1-based immune checkpoint inhibitors are a good example of a class of agents that have a relatively flat dose-response relationship.

The recognition of the uncoupling of dose intensity and outcomes for some cancer agents led the FDA Oncology Center of Excellence to launch Project Optimus, an initiative to improve the process of selecting appropriate doses and schedules of oncology drugs.^1,2^ Goals for the Project include communicating expectations, providing opportunities to meet with FDA regarding appropriate clinical design, as well as guidance to find the best dose and schedule of an agent to give to an individual patient.^2,3^

Tools to achieve the goals of Project Optimus may include patient-reported outcome data, quality-of-life assessments, or reporting of the frequency of a specific or of any adverse event. However, we note that while there are well-defined methods in survival analysis and radiologic analysis to define efficacy, there is no widely accepted method to summarize and compare the investigator-evaluated toxicity and benefit of 2 treatments, or different doses/schedules of the same treatment.

We previously developed a simple summary toxicity metric, the weighted toxicity score (WTS), which represents the total toxicity burden observed in one arm of a randomized clinical trial.^4^ The WTS method involves condensing all Common Terminology Criteria for Adverse Events (CTCAE) grades 1 to 4 maximum toxicities into a single metric by totaling the product of every grade × frequency of toxicities in a clinical trial toxicity table. The WTS initially was calculated for placebo-controlled clinical trials of oral kinase inhibitors, in order to separate the effects of the cancer from the effects of the drug. The WTS and a clinical metric, such as progression-free survival or response rate, can be graphed as a data pair or compared as differences or ratios, in order to inform patients of the cost-benefit expectations for different treatments.

In this work, we extend the WTS to clinical trials involving immunotherapy and chemoimmunotherapy for lung cancer to show that this simple method can be applied to the context beyond oral kinase inhibitors. We then apply the metric to trials highlighted in the Project Optimus paper noted above that identified successful examples of the optimization of cancer therapy dose and schedule. We show that these examples open the door to employ the WTS or other summary toxicity metrics to more easily visualize and ascertain an optimal dose and schedule of a specific cancer treatment.

## Methods

### Trial Selection

Our goal was to confirm the utility of the WTS metric in new clinical trial contexts to measure toxicity of separate arms of a trial. Previously, we examined the benefit/toxicity ratio via the WTS metric in a series of placebo-controlled randomized trials of oral kinase inhibitors. Here, we examined randomized trials of immunotherapy agents tested against standard of care chemotherapy in lung cancer (predominantly non-small cell lung cancer [NSCLC]). The second group of studies examined were those cited in Shah et al,^1^ which were put forward as examples of trials that emphasize the goals of the FDA’s Project Optimus. These studies largely examined outcomes for patients treated at lower than the FDA approved dose from prior studies.

In the first setting, we collected studies by literature search of PubMed for randomized controlled trials in lung cancer between January 2010 and December 2020. We then selected studies employing PD-1/PD-L1-based agents, as immune checkpoint inhibitors (ICI) as a therapy appear to be less toxic than chemotherapy.^5^ Studies were categorized based on the number of arms of the trial, and whether they examined ICI alone or in combination with chemotherapy. Trials using ICI with radiation therapy were excluded, given our desire to isolate and compare toxicities associated with various systemic therapies only. We identified 9 trials comparing immunotherapy alone to chemotherapy alone, 10 trials comparing immunotherapy in combination with chemotherapy to chemotherapy alone, and three 3-arm trials that compared varying doses or immunotherapy combinations to chemotherapy alone.

For the second setting, trials cited in reference 1 were investigated, because they were cited as successful examples of trials examining the optimization of cancer therapy dose and schedule. We again only examined trials that were both comparative and reported sufficient data on efficacy and adverse events (AE) by which we could calculate WTS. From these trials, we identified 4 for which a WTS could be calculated.

The studies we selected are listed in Supplementary Table S1, with references.

### WTS Calculations

**Figure 1.**
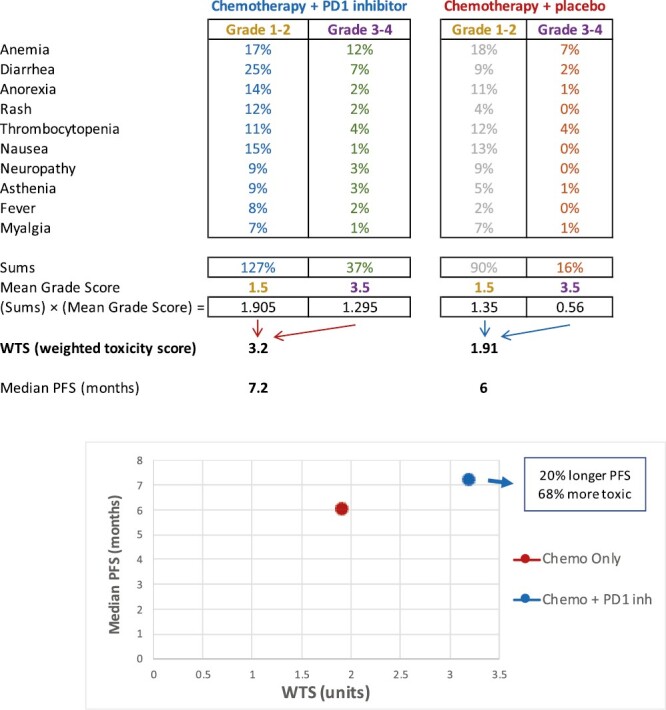
(**A**) Example: calculation of the weighted toxicity score (WTS). In this example, the grade I-II and grades III-IV toxicities were collapsed into single percentages. As a result, the weighting for grade I-II toxicities is (1 + 2)/2 = 1.5 and the weighting of grade III-IV toxicities is (3 + 4)/2 = 3.5. 2 treatments are compared. For each treatment, the sum of the frequency of all grade I-II or III-IV toxicities is multiplied by the mean grade score (1.5 or 3.5). The sum of those weighted toxicities defines the WTS, which for chemotherapy + placebo is 1.91 and for chemotherapy + PD1 inhibitor 3.2. Thus, the combination chemoimmunotherapy treatment has 20% longer median progression-free survival (PFS) and is 68% more toxic than chemotherapy alone. **(B)** Each (WTS, outcome) data pair is graphed, allowing direct comparison of the outcome and the summary toxicity in one figure.

As previously defined, the WTS is the sum of products of each Common Terminology Criteria for Adverse Events CTCAE grade I to IV toxicity and the frequency of that specific toxicity, as described in a clinical trial toxicity table.^4^ This simple weighting scheme provided the lowest variance in comparison to other weighting schedules for grade I to IV toxicities that we examined. In studies where reported toxicity grades were combined (eg, grade I and II given as a single proportion of patients), the mean of the weighted toxicity values (as though the AEs were grade 1.5) was used. For each identified study, a WTS was calculated for each trial arm, which allowed for the simultaneous comparison of toxicity and efficacy in the clinical trial as a data pair that can be graphed on a 2-dimensional plot of clinical outcome versus WTS as abscissa and ordinate. Ratios and differences of WTS and clinical parameters such as PFS between study arms were also generated. An example of calculating the WTS using a maximum toxicity table is provided in Fig. 1.

## Results

### Lung Cancer: Aggregate Data

We identified 22 trials of FDA-approved immune checkpoint inhibitors in comparative, randomized studies of lungs cancer with sufficient AE data to calculate the WTS. Immunotherapy in these studies was examined in combination with or compared to chemotherapy (Supplementary Table S2). There were variations in how toxicity was reported with AE inclusion cutoffs being described for each study in this table. These summary data provide an understanding of the range and variability of the WTS in and across studies.

For the first category of trials that compared immunotherapy alone to chemotherapy alone, immunotherapy was consistently less toxic than chemotherapy by WTS, with chemotherapy on average 3.46 times as toxic as immunotherapy. WTS for the immunotherapy arms ranged from 1.03 to 4.28 (mean 1.87, median 1.69), while WTS for the chemotherapy arms was consistently higher than the immunotherapy arms, from 4.60 to 7.26 (mean 5.67, median 5.63). Efficacy data are also presented in Supplementary Table S2 and showed consistent superiority of median PFS for chemotherapy, but superior overall survival with immunotherapy, which was already well recognized from the individual trial data.

We also reviewed the set of trials that compare chemoimmunotherapy combinations to chemotherapy alone (Supplementary Table S2). Chemoimmunotherapy combinations were more toxic than chemotherapy alone, shown by the larger WTS within the combination arms, which in aggregate were 1.12 times as toxic than the chemotherapy alone. WTS for the chemotherapy-immunotherapy combination arms ranged from 2.63 to 13.84 (mean 8.43, median 8.04), while WTS for the chemotherapy alone arms ranged from 2.48 to 12.94 (mean 7.38, median 7.32). Note that these WTS are higher than that for immunotherapy alone in nearly every case as well. Chemoimmunotherapy combinations consistently outperformed the chemotherapy arms on both metrics of efficacy (median OS and median PFS).

### Lung Cancer: Individual Study Data

**Figure 2.**
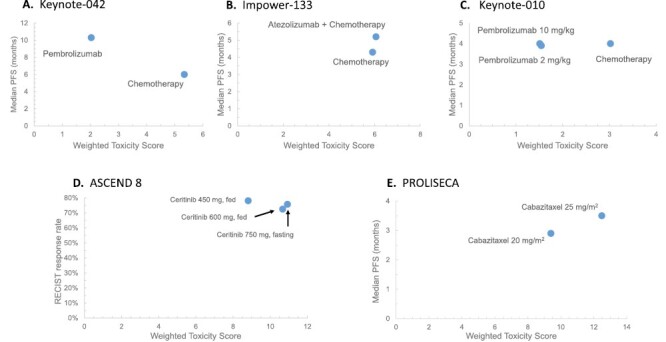
Example WTS comparisons for individual lung cancer trials as well as for studies comparing trials with dosing less intensive than regulator approved doses/schedules. (**A**) immunotherapy vs chemotherapy: Keynote-042, comparing pembrolizumab to chemotherapy by WTS and median PFS in NSCLC; (**B**) immunotherapy ± chemotherapy combination vs chemotherapy in NSCLC: Impower-133 comparing Atezolizumab + chemotherapy to chemotherapy by WTS and median PFS; (**C**) 3-arm trial: Keynote-010: comparing pembrolizumab 2 mg/kg and Pembrolizumab 10 mg/kg to chemotherapy by WTS and median PFS. **(D)** ASCEND 8, comparing Ceritinib 450 and 600 mg (fed state) to Ceritinib 750 (fasting) by WTS and RECIST response rate; **(E)** PROLISECA, comparing Cabazitaxel 25 vs 20 mg/m^2^ by WTS and median PFS.

Arguably more useful than the aggregate data, the WTS provides granular data to discuss a specific clinical setting with a patient. With the Keynote-042 study of chemotherapy versus pembrolizumab for PD-L1(+) NSCLC as an example,^6^ pembrolizumab was both 62% less toxic and 71% or 64% more efficacious when measuring improvement by median PFS or median OS, respectively. These advantages are shown graphically in Fig. 2A, where toxicity is summarized into a single scalar to help visually represent the (PFS or OS, WTS) data pair. Specifically, pembrolizumab was less toxic (WTS of 2.03 vs 5.34) and more efficacious (median PFS of 10.3 vs 6.0 months; median OS of 20.0 vs 12.2 months). Conversely, from an example in small cell lung cancer, the difference was modest. In the Impower-133 trial of carboplatin-etoposide ± atezolizumab in Fig. 2B, the combination regimen has modestly greater toxicity and efficacy compared to chemotherapy by itself WTS of 6.06 vs 5.91, and median PFS 5.2 months versus 4.3 months. Consistent with the original study,^7^ the WTS is consistent with the study conclusion of the benefit of adding atezolizumab to standard chemotherapy with negligible differences in toxicity.

The WTS is also easily applied to 3 arm trials, as in the Keynote-010 trial of pembrolizumab (2 different doses) versus docetaxel for previously treated PD-L1 NSCLC^8^ in Fig. 2C, where the metric allows for a simultaneous comparison of all 3 arms. Using this index, pembrolizumab 2 mg/kg yielded a WTS of 1.54, pembrolizumab 10 mg/kg yielded a WTS of 1.51, and docetaxel yielded a WTS of 3.02. In this study, there was no significant difference in median PFS between groups; however, median OS for both immunotherapy groups were significantly longer than the chemotherapy arm (10.4 and 12.7 vs 8.5 months, respectively). Again, the data generated using this index support the study’s primary conclusion^8^ and quantitates and allows visualization of the toxicity difference between study arms.

### Project Optimus: Dose Comparisons

We further applied the WTS to assess toxicity of varying doses of an FDA-approved anti-cancer agent. Shah et al^1^ identified a number of trials where postapproval dose finding was performed, which provided an ideal opportunity to test this metric. Many of these trials were conducted due to substantial side effects at the regulator-approved starting dose, which suggested a need for further dose finding. For these trials, we calculated WTS for applicable studies in Shah et al. In each case, data generated from the WTS supported the conclusions found in the separate investigations, and summarized toxicity in a simple and visually clear manner.

For example, in the ASCEND 8 trial,^9^ ceritinib had been approved at a 750 mg daily dose, but a subsequent study was conducted to determine if the side effects associated with that agent could be reduced. Ceritinib was evaluated at 3 different doses and ultimately ceritinib 450 mg with food prevailed, as it was shown to have consistent efficacy and less gastrointestinal toxicity. Assessing the WTS and efficacy values below in Table 1 and Fig. 2D, these differences are visualized. Ceritinib 450 mg in 2-way comparisons with the 600 mg and 750 mg formulations demonstrated approximately the same response rate (78% vs 73% and 76%, respectively) and had less toxicity as calculated by our WTS metric (8.8 vs 10.67 and 10.93 respectively), all consistent with the conclusions from the primary study.

**Table 1.** Pairwise comparisons of WTS and efficacy for different arms of individual dose comparison trials supporting lower than approved dose/schedule of anti-cancer agents.

Dosing trials from Shah et al.									
Trial/arm	Diagnosis	AE inclusion cutoff	Efficacy measure	Arm A	WTS Arm A	Efficacy Arm A	Arm B	WTS Arm B	Efficacy Arm B
ASCEND 8, Arm 1 vs 2	NSCLC	20%	RECIST overall response rate	Ceritinib 450 mg with food	8.81	78%	Ceritinib 600 mg with food	**10.67**	73%
ASCEND 8, Arm 1 vs 3	NSCLC	20%	RECIST overall response rate	Ceritinib 450 mg with food	8.81	78%	Ceritinib 750 mg, fasting	**10.93**	76%
ASCEND 8, Arm 3 vs 3	NSCLC	20%	RECIST overall response rate	Ceritinib 600 mg with food	10.67	73%	Ceritinib 750 mg, fasting	**10.93**	76%
CAA100834 Arm 1 vs 2	Chronic-phase CML	10%	Hematologic/Cytogenic Response	Dasatinib 100 mg once daily	8.60	90%/ 41%	Dasatinib 50 mg twice daily	**9.83**	92%/42%
CAA100834 Arm 1 vs 3	Chronic-phase CML	10%	Hematologic/cytogenic response	Dasatinib 100 mg once daily	8.60	90%/ 41%	Dasatinib 140 mg once daily	**10.65**	86%/44%
CAA100834 Arm 1 vs 4	Chronic-phase CML	10%	Hematologic/cytogenic response	Dasatinib 100 mg once daily	8.60	90%/ 41%	Dasatinib 70 mg twice daily	**10.64**	87%/45%
CAA100834 Arm 2 vs 3	Chronic-phase CML	10%	Hematologic/cytogenic response	Dasatinib 50 mg twice daily	9.83	92%/ 42%	Dasatinib 140 mg once daily	**10.65**	86%/44%
CAA100834 Arm 2 vs 4	Chronic-phase CML	10%	Hematologic/cytogenic response	Dasatinib 50 mg twice daily	9.83	92%/ 42%	Dasatinib 70 mg twice daily	**10.64**	87%/45%
CAA100834 Arm 3 vs 4	Chronic-phase CML	10%	Hematologic/cytogenic response	Dasatinib 140 mg once daily	10.65	86%/ 44%	Dasatinib 70 mg twice daily	**10.64**	87%/45%
Optic Arm 1 vs 2	Chronic-phase CML	10%	12 month MR2 rate	Ponatinib 15 mg	6.81	24%	Ponatinib 30 mg	**7.63**	28.00%
Optic Arm 1 vs 3	Chronic-phase CML	10%	12 month MR2 rate	Ponatinib 15 mg	6.81	24%	Ponatinib 45 mg	**9.07**	42.00%
Optic Arm 1 vs 3	Chronic-phase CML	10%	12 month MR2 rate	Ponatinib 30 mg	7.63	28%	Ponatinib 45 mg	**9.07**	42.00%
PROSELICA Arm 1 vs 2	Prostate cancer	N/A	Median PFS	Cabazitaxel 20 mg/m2	9.40	2.90	Cabazitaxel 25 mg/m2	**12.49**	3.50%

ASCEND 8 Arm 1 = ceritinib 450 mg (fed state); ASCEND 8 Arm 2 = ceritinib 600 mg (fed state); ASCEND 8 Arm 3 = ceritinib 750 mg (fasting); CA100834 Arm 1 = dasatinib 100 mg daily; CA100834 Arm 2 = dasatinib 50 mg twice daily; CA100834 Arm 3 = dasatinib 140 mg once daily; CA100834 Arm 1 = dasatinib 70 mg twice daily; OPTIC Arm 1 = ponatinib 15 mg; OPTIC Arm 2 = ponatinib 30 mg; OPTIC Arm 3 = ponatinib 45 mg; PROLISECA Arm 1 = cabazitaxel 20 mg/m^2^; PROLISECA Arm 2 = cabazitaxel 25 mg/m^2^.

Note: Dark red indicates WTS ratio (more toxic/less toxic arm) >2*x*; red, 1.51 − 1.99*x*; orange, 1.26 − 1.50*x*; yellow, 1.11 − 1.25*x*; gray, 1 − 1.1*x.* A color code, also used in Supplementary Table S2, is used to highlight the degree to which one arm of the trial is more toxic than the other.

Abbreviations: CML: chronic myeloid leukemia; NSCLC: non-small cell lung cancer; MR2: molecular response with ≤1% BCR-ABL; WTS: weighted toxicity score.

In the PROLISECA trial as a further example,^10^ the WTS metric demonstrates graphically (Fig. 2E) each arm’s toxicity relative to the other. Cabazitaxel was initially approved at 25 mg/m^2^, but at this approved dose, there were significant hematologic and infectious adverse events. The similar benefit and decreased toxicity observed with the 20 mg/m^2^ dose are made evident examining the (outcome, WTS) data pair for each dose.

## Discussion

The assessment and management of a patient’s AEs is one of the most important issues a medical oncologist has to address. Given the high rate of dose reduction from FDA-approved doses in cancer for many oral kinase inhibitors, we hoped that a better way to discuss summary toxicity would make conversations about cost and benefit of different treatments more transparent. For example, whether an agent has a 5% lower median progression-free survival but 25% less toxicity can help inform whether to use higher or lower doses of an agent for a specific patient. As of May 2023, FDA is asking for comments on guidance to expand dose finding in early phase clinical trials of novel anti-cancer agents.^2^

In prior and present work, we demonstrated that the WTS metric is easily calculated and provides useful and consistent data across clinical trials with multiple types of systemic therapies. Most importantly, the summary score yields results that are consistent with the primary trial outcomes. We also note that the WTS works regardless of how the data are presented (eg, clustering grades 1-2 and 3-4 toxicities instead of reporting the frequency of each event for each grade, as we show in the example in Fig. 1), as the WTS treats the groups of patients within the trial similarly, as an internal control. The internal control provided by randomization is a key strength of any comparative toxicity metric, including the WTS and is expected to dilute any effects of different clinician evaluations of toxicity over time, or other reporting issues such as cutoffs for toxicity reporting, or merging of different grades of toxicity, especially if the sample size is sufficiently large.

There are a variety of limitations of the WTS to consider, which make this metric one starting point for the assessment of overall toxicity in a cancer clinical trial. The toxicity tables presented in publications represent the worst overall toxicity on the trial and do not reflect the lower degree of toxicity if the dose or schedule of the medication are modified. More complex summary toxicity metrics that include the duration of the toxicity at a specific grade are another way of summarizing cancer treatment toxicity data. Examples of these and other value frameworks where radiologic or clinical outcomes are tempered by the degree of toxicity of the therapy are highlighted in Table 2. Some of these metrics have evaluated randomized clinical trials data retrospectively, when patient-level data are available. In one such evaluation, cumulative toxicity was lower the longer that patients stayed on therapy, although the ratio of summary toxicity score early or later in a trial were similar.

**Table 2.** WTS and other summary toxicity metrics or outcome measures (value frameworks) in which toxicity can modify the relative benefit of treatment.

Year of publication	First author(s) and reference	Name of Metric (acronym)	Elements of metric	Patient level data required	All toxicities of certain grade considered equally problematic	Demonstration of use at the patient level in a clinical trial?
Examples of derived toxicity measures that are independent of clinical or radiological outcomes						
1996	Gelber^11^	Q-TWiST (toxicity component)	Toxicity score as a function of time, arbitrarily chosen in the initial description of the model, often 0.5 on a 0-1 scale.	No	Yes	No, not applicable
2012, 2018	Lee^12,13^	Toxicity burden score (TBS)	Clinician or patient assessment of severity of class of adverse event providing a intensity multiplier for the frequency of severity of a given adverse event	Yes	No	Yes, retrospective
2018, this manuscript	Carbini^4^	WTS)	Frequency of toxicity, highest grade of toxicity	No	Yes	No, not applicable
2021, 2022	Razaee,^14^ Langlais^15^	TI (incorporating maximum grade and frequency of lower grade toxicities)	Number of toxicities at each grade. Asymptote of TI is the grade of the worst toxicity + 1	Yes	Yes	Yes, retrospective with clinician-reported outcomes and with patient-reported outcomes
2021	Ruppert^16^	Adverse event burden score (AEsc)	Frequency of toxicity, grade of toxicity, duration of toxicity at that grade	Yes	Yes	Yes, retrospective
2021	Lopes^17^	Onset time and adverse event load	Grade of adverse event, duration, highest grade	Yes	Yes	Yes, retrospective
Examples of derived outcomes measures that use toxicity data to modify clinical or radiological outcomes						
2015, 2016	Schnipper^18,19^	ASCO Value Framework	Survival or radiological outcome score is modified by degree of toxicity based on frequency of grade 1-2 vs grade 3-4 events and persistence of over 1 year	No	Yes	No, not applicable
2016, 2017	Cherny^20,21^	ESMO-MCBS)	Survival or radiological outcome score is modified by frequency of severe toxicity	No	Yes	No, not applicable

Some of the metrics use patient level data, in order to include the duration of the adverse event at a specific grade.

Abbreviations: TBS: toxicity burden score; WTS: weighted toxicity score; TI: toxicity Index; ESMO-MCBS: European Society for Medical Oncology Magnitude of Clinical Benefit Scale.

We also note that the inherent problems of comparing data across studies in terms of outcomes applies to an even greater extent in toxicity evaluation, for example, in such realms as severity or attribution to treatment or diagnosis. Accordingly, the WTS should not be used to compare toxicity across different trials. Finally, the WTS calculation provides no specific measure of variability. That said, a large number of observations (number of individual patient visits on a study) are required to assemble toxicity tables, as some measure of robustness of those data. In that sense, a study with *n* = 100 will be less reliable than a study with *n* = 1000 patients, for example.

There are a number of applications for the WTS or other cumulative toxicity metrics. An important potential use of the WTS is to apply it prospectively while a trial is underway, to help investigators to identify excessive cumulative toxicity while the study is ongoing, and thus trigger dose reductions for individuals or groups of patients. The data could also be used by data safety monitoring boards to help make decisions about toxicity of a specific treatment in a clinical trial. A major limitation of such an analysis is the need for complete data to compare the 2 or more treatments accurately. In addition, the WTS can be applied to patient-reported outcome data as well, because some systems such as the PRO-CTCAE already have a grading system by which summary metrics can be constructed in a similar manner. Work to apply to the PRO-CTCAE in a longitudinal manner during a clinical trial to enumerate patient cumulative toxicity burden for a specific patient have been described, which leverage this important type of toxicity data.^15^

In sum, we have generalized the use of the WTS developed in clinical trials using kinase inhibitors to cancer clinical trials involving chemotherapy and immunotherapy. In future work, we will apply this metric to guide dose selection of novel and approved agents in future cancer clinical trials prospectively, in order to realize some of the goals of Project Optimus.

## Supplementary Material

oyad192_suppl_Supplementary_Table_S1

oyad192_suppl_Supplementary_Table_S2

## Data Availability

The data underlying this article will be shared on reasonable request to the corresponding author.
